# Liver Steatosis: A Marker of Metabolic Risk in Children

**DOI:** 10.3390/ijms23094822

**Published:** 2022-04-27

**Authors:** Costanza Renata Neri, Serena Scapaticci, Francesco Chiarelli, Cosimo Giannini

**Affiliations:** Department of Pediatrics, University of Chieti, Via dei Vestini, 5, 66100 Chieti, Italy; costanzanerix@gmail.com (C.R.N.); serena.scap91@gmail.com (S.S.); cosimogiannini@hotmail.it (C.G.)

**Keywords:** NAFLD and children, NASH and children, pediatric MAFLD, Metabolic Syndrome (MetS) and children, hepatic fibrosis in children

## Abstract

Obesity is one of the greatest health challenges affecting children of all ages and ethnicities. Almost 19% of children and adolescents worldwide are overweight or obese, with an upward trend in the last decades. These reports imply an increased risk of fat accumulation in hepatic cells leading to a series of histological hepatic damages gathered under the acronym NAFLD (Non-Alcoholic Fatty Liver Disease). Due to the complex dynamics underlying this condition, it has been recently renamed as ‘Metabolic Dysfunction Associated Fatty Liver Disease (MAFLD)’, supporting the hypothesis that hepatic steatosis is a key component of the large group of clinical and laboratory abnormalities of Metabolic Syndrome (MetS). This review aims to share the latest scientific knowledge on MAFLD in children in an attempt to offer novel insights into the complex dynamics underlying this condition, focusing on the novel molecular aspects. Although there is still no treatment with a proven efficacy for this condition, starting from the molecular basis of the disease, MAFLD’s therapeutic landscape is rapidly expanding, and different medications seem to act as modifiers of liver steatosis, inflammation, and fibrosis.

## 1. Introduction

Non-Alcoholic Fatty Liver Disease (NAFLD) has become one of the most common forms of chronic hepatic disease over the last years, both in the adult and the pediatric populations [1,2,3]. Its wide dissemination over the last decades has made NAFLD one of the actual biggest global issues [4]. The term NAFLD describes a continuum spectrum of progressive and partially reversible liver damages. In fact, its first evidence is characterized by simple steatosis, defined as triglycerides (TGs) accumulation in more than 5% of hepatocytes or a fat fraction >5.6% assessed by proton magnetic resonance spectroscopy (HMRS) [5,6]. Thereafter, the persistence of risk factors induces the progression to a further stage characterized by lobular inflammation and the different degrees of fibrosis that define non-alcoholic steatohepatitis (NASH) in the absence of secondary causes of liver injury and excessive alcohol consumption [5,6]. If untreated, the natural course of the disease evolves towards end-stage liver disease (cirrhosis) and hepatocarcinoma (HCC) later in life, although there is also growing evidence that HCC can develop in a fatty liver in the absence of cirrhosis [5,7].

A strong relationship between hepatic steatosis, Insulin Resistance (IR), and Metabolic Syndrome (MetS) has been pointed out, the latter featured by the association of central obesity, impaired glucose tolerance, dyslipidemia, and hypertension [8]. To stress the strict connection between NAFLD and metabolic dysfunctions, this condition has been recently renamed both in children and adults as Metabolic Dysfunction Associated Fatty Liver Disease (MAFLD), which seems more representative of the disease etiology and pathogenesis [9,10]. While a NAFLD diagnosis requires an exclusion of other causes of hepatic steatosis, a MAFLD diagnosis implies the detection of liver steatosis by imaging techniques, by blood biomarkers or scores, or by liver histology, in addition to one of the parameters of metabolic dysfunction [8]. Although this new definition does not fully explain the wide spectrum of the disease [11], it might offer the possibility to detect people with an unfavorable metabolic profile and a higher risk of progressing to end-stages liver damage [12,13].

Therefore, a greater knowledge of the disease is necessary to detect individuals at risk for MAFLD development precociously. The scientific interest is focused on the research of all the factors implicated in the onset of this intricated spectrum of liver damages in order to implement prevention and intervention strategies. For this reason, we will discuss the current knowledge about epidemiology, risk factors, and the recent theories about the pathogenesis of MAFLD. In the last part of the manuscript, we will summarize the main knowledge on the currently available methods and potential therapeutical and prevention strategies influencing the natural history of the disease. Particularly, the molecular connections between potential therapeutic strategies and both pathogenesis and complications will be explored.

## 2. Methods

We reviewed the literature analyzing the complex dynamic behind the development of liver steatosis and metabolic dysfunctions. We present results from systematic reviews and meta-analyses, randomized controlled trials (RCTs), and large observational studies. We performed a PubMed search by topics and/or relevant authors up to April 2022 of the adult and pediatric literature on pediatric liver steatosis using the following keywords: NAFLD and children, NASH and children, pediatric MAFLD, Metabolic Syndrome (MetS) and children, and hepatic fibrosis in children.

## 3. Epidemiology

MAFLD is a disease of global interest affecting individuals of all ages and ethnicities whose great extension is directly related to the increased incidence of obesity worldwide, even in the youngest. Nowadays, obesity is one of the most worrying public health problems, involving millions of children in developed countries [14]. In fact, changes in food habits and a sedentary lifestyle, especially during the actual pandemic, have dramatically influenced the global incidence and prevalence of non-communicable diseases in the pediatric population, including obesity and NAFLD [15]. The most recent data provided by the Centers for Disease Control and Prevention (CDC) reported a worldwide prevalence rate of overweight and obesity among children and adolescents aged between 2 and 19 years equal to 19% in the two-year period of 2017 and 2018, with an increase of roughly 1% compared to 2016 [16]. Globally, teenagers aged 12–19 years are the most affected (21.2%), with a downwarded trend paralleling the decreased age (prevalence of 20.3% and 13.4% among 6–11 year olds and 2–5 year olds, respectively) [16]. The most alarming data is that 39 million children under the age of 5 were overweight or obese in 2020, being therefore exposed to a major risk of complications [17]. Concurrently, the rate of NAFLD in the pediatric population has doubled in the last 20 years, rising from 3.9% in 1988–1994 to 10.7% in 2007–2010 [18]. Globally, NAFLD and NASH prevalence were augmented from 19.34 million in 1990 to 29.49 million in 2017 in young people, with an incidence that increased to 1.35 [19]. Therefore, this disease should not be considered mainly specific to adulthood, as was formerly thought. Among all the metabolic diseases, obesity confers the higher risk of NAFLD development, with an increased prevalence rate of 20.23% (95% CI 12.87–30.33) in overweight and 38.47% (95% CI 29.75–48.00) in obese children and adolescents within the general population [20]. 

Epidemiological data referring to the different diagnostic criteria of MAFLD and NAFLD are not unique. A study from the US NHANES III (1988–1994) database showed that the prevalence of MAFLD was lower than that of NAFLD (31.24% vs. 33.23%, *p* < 0.05) [13]. In contrast, based on the Jinchang cohort that included 30,633 participants, the prevalence rates of MAFLD and NAFLD were attested as 21.03% and 18.83%, respectively [21].

Nowadays, MAFLD’s global prevalence is near to 45% in those settings based on child obesity clinics and 34% in the general population among overweight or obese children and adolescents aged between 1 and 19 years, independently of the diagnostic technique used [20]. The COVID-19 pandemic has contributed to the further increase in incidence of MAFLD. In fact, in order to prevent SARS-CoV-2 dissemination, measures such as social distancing, stay-at-home orders, and school closure have been implemented worldwide, reducing the possibility to practice physical activities [22]. Likewise, quarantine measures have led to changes in food habits and eating patterns, with an increased income of junk food and lower adherence to the Mediterranean diet [23]. As a consequence, an increased rate of weight gain has been reported during the SARS-CoV-2 pandemic, with a consequent increment of health problems associated with childhood obesity, including NAFLD [24]. However, fatty liver disease prevalence is extremely heterogeneous, depending on multiple factors. One of the reasons that may explain the variability in the prevalence rate in the adult population lies in the lack of a simple noninvasive diagnostic test [25]. Nevertheless, when the MAFLD diagnosis is based on biochemical tests and an ultrasound evaluation, early studies reported an overall prevalence of 3–7% among children and adolescents [26].

**Figure 1:**
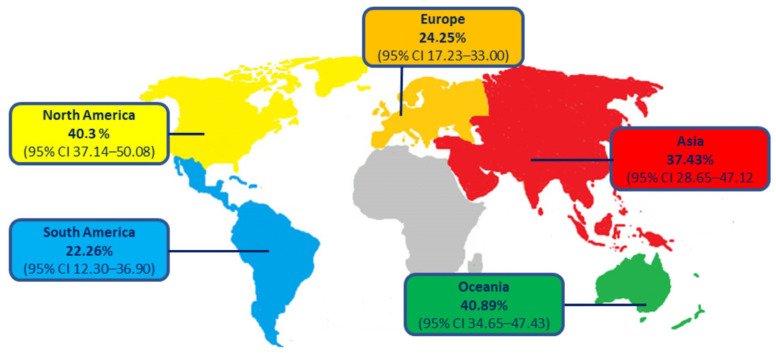
Estimated global prevalence of pediatric MAFLD in overweight or obese children and adolescents.

Moreover, epidemiological data are strongly influenced by the country of origin, with a prevalence of MALFD equal to 43.50% in North America, even if the greatest increases were observed in North Africa and the Middle East [20,27] (Figure 1). 

Besides epidemiological differences existing between individuals living in different areas of the world, large multi-ethnic cohort studies have reported an interethnic variability in MAFLD susceptibility, with a high risk in Hispanics, moderate in Europeans, and low in African-Americans, independent from adiposity, IR, and socioeconomic factors [28]. These data seem to be surprising, considering that African-Americans tend to have higher levels of IR than Caucasians and Hispanics. The explanation given by researchers is that MAFLD development and progression is not necessarily associated with just few risk factors (obesity, IR, and MetS), but probably, MAFLD susceptibility relies on the different individual metabolic states. Particularly, it has been shown that African-Americans tend to accumulate a minor amount of visceral than subcutaneous fat, with a lower tendency of developing hepatic steatosis (see paragraph ‘NAFLD and metabolic dysregulation’ for more details) [28]. To date, children of Hispanic (11.8%) and Asian (10.2%) ethnicities have a higher prevalence of MAFLD compared to Caucasian children (8.6%) [29]. The different expressions of the risk allele of the Patatin-like phospholipase domain-containing protein 3 (PNPLA3) gene among the various ethnic groups seems to explain some of this ethnic variability, since it is more represented in Hispanics (49%), followed by non-Hispanic Whites (23%) and African Americans (17%) [30]. However, the reasons why ethnicity differences exist are not completely known. In this regard, the complex interplay between genetic predisposition and environmental factors might help us to understand the mechanisms behind these epidemiological discrepancies.

**Figure 2:**
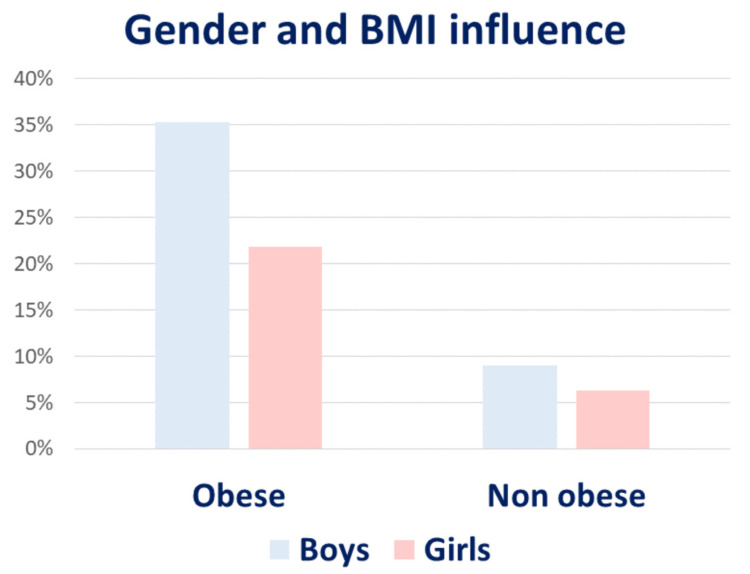
Difference in prevalence according to gender and BMI in children and adolescents.

Interestingly, gender can also influence MAFLD prevalence. Recent data provided by International Literature describe a rate of MAFLD almost twice among male children and adolescents compared to female children, with a progressively rising prevalence according to greater Body Mass Index (BMI) values (35.3% vs. 21.8% in obese boys vs. girls) [29,31]. Apparently, differences in adiposity, metabolic risk factors, and body fat distribution (which tend to shift towards abdominal obesity after menopause) could partially explain these results [8] (Figure 2).

## 4. Genetic Risk Factors

The susceptibility to the development of NAFLD cannot be conferred exclusively to metabolic factors. Epidemiological differences existing within people belonging to different ethnicities or living in different countries could be indicative of genetic and epigenetic implications in NAFLD susceptibility and development, especially since not all children with obesity develop NAFLD [32]. In this regard, a lot of genetic variants associated with hepatic steatosis have been recently described, and the research on this topic is in continuous progress [29]. The exposition to a dysmetabolic environment fails to explain the wide variability of the risk of the development and progression of MAFLD existing between people living in the same region exposed to the same environmental factors. Furthermore, the presence of a familiar cluster of MAFLD has been reported, presuming a hereditable nature of the disease [29]. In this regard, the scientific community has been focused on researching a specifically genetic background involved in liver fat accumulation, inflammation, and consequently hepatic injury that eventually could be clinically used for risk stratification and personalized care [29,33]. Although different sources have suggested a genetic involvement in childhood onset of the disease [34,35], genetic variants might also be implicated in the different expressiveness and severities of the disease. Twin studies recognized that half of the variability in alanine aminotransferase (ALT) values and fat contents depend on hereditability factors and that liver steatosis and fibrosis are joined by a genetic compound [34,36]. The scientific attention was primarily focused on genes playing a role in lipid handling, insulin signaling, oxidative stress, or hepatic fibrogenesis. The knowledge in this field has expanded thanks to a genome wide-association study (GWAS) that allows to search different genetic variants associated with the NAFLD phenotype simultaneously. Unfortunately, very few studies have been conducted involving the pediatric population and the large part of the information currently available derived from adult population investigations. Different scientific analyses carried on pediatric and adult individuals have identified common genetic variant drivers for lipidic profile alterations and, subsequentially, hepatic fat accumulation [30,37,38]. Among the polymorphisms analyzed in all ethnic groups, the rs738409 C > G single-nucleotide polymorphism that results in the I148M protein variant of PNPLA3 is the first best-studied factor of susceptibility [30]. The gene encodes a protein expressed on droplet surfaces in hepatic cells, intimately involved in lipid remodeling. The presence of a structural and functional protein alteration negatively affects the normal liver fat balance, predisposing it toward hepatic steatosis. Furthermore, an impaired release of retinol from hepatic stellate cells has been described in association with the I148M protein, which may represent the trigger event to inflammation and fibrosis [30]. Therefore, the I148M PNPLA3 variant is associated not only with MAFLD but also with its progression towards terminal stages [29,30]. From an epidemiological point of view, it has been found in individuals of all ages, even if the major susceptibility toward NAFLD development has been observed in children under 18 years. It is more frequent in Hispanics, in which is it responsible for about 50% of the risk of NAFLD development [30].

Another gene involved in MAFLD risk is transmembrane 6 superfamily member 2 (TM6SF2), in which the rs58542926 C > T single-nucleotide polymorphism encodes the E167K protein. The resulting genic product alters the normal very-low density lipoprotein (VLDL)-mediated lipid secretion favoring hepatic fat accumulation both in adults and children [29,37,39,40,41]. Clear evidence of the importance of regulation of lipid metabolism derives also from the correlation existing between MAFLD and variants of the genes glucokinase regulator (GCKR) and membrane bound O-acyl transferase 7 (MBOAT7). The first one encodes for a regulator of glucokinase, a key enzyme of lipogenesis. The consequence of its mutation conducts to a reduced response to inhibitory stimulus increasing lipid production. Moreover, the lower MBOAT7 gene expression is associated with alterations in phospholipids remodeling, phenomenon which can lead to secondary fat accumulation. However, although these last two gene variants have been confirmed as associated with increased risk of NAFLD in adults, there are still poor data in children. Unlike the polymorphisms so far discussed, a variant of the gene encoding protein phosphatase 1 regulatory subunit 3B (PPP1R3B) has been described as protective against NAFLD development as a consequence of a reduced DNL and increases glycogen synthesis. Nevertheless, also for this gene there is no evidence of a certain etiologic role in pediatric NAFLD.

Other interesting perspectives in terms of genetic characterization derives from an autoptic study conducted on 234 Hispanic boys focused on the aim to find exploring new possible allelic variants [42]. Among these, trafficking protein particles complex 9 (TRAPPC9) have been associated with NAFLD activity score (NAS). While a single-nucleotide polymorphism in a region close to actin-related protein 5 seems to be related to fibrosis [42]. Additionally, rare genetic mutations in genes involved in NAFLD pathogenesis have been described presuming a role of genetic predisposition. Apolipoprotein B mutation seems able to modify the hepatic distribution of fats leading to a progressive form of NAFLD [43]. Furthermore, genetic alteration in reverse transcriptase gene telomere (TERT) might promote telomer shortening and cell senescence driving the hepatic disease towards the terminal stages [44,45].

It is well established that genetic variants and/or mutations have an important role in the MAFLD risk determination. It has been proposed an additive effect of different genetic alterations in increasing NAFLD appearance, with the possibility to draw-up a genetic risk score to stratify the risk of NAFLD development [38,46]. In this regard, Zusi et al. evaluated the association between NAFLD and eleven single-nucleotide polymorphisms (SNPs) at genetic loci potentially associated with liver damage (GCKR, MBOAT7, and GPR120); oxidative stress (SOD2); lipid metabolism (PNPLA3, TM6SF2, LPIN1, ELOVL2, FADS2, and MTTP); and fibrogenesis (KLF6) in a pediatric population. The aim of the study was to obtain a genetic risk score (GRS) considering both these SNPs and clinical risk factors. The authors were able to show that the combination of a 11-polymorphism GRS to known clinical risk factors (ethnicity, weight gain, and insulin resistance) significantly improved the possibility of establishing a real risk of developing NAFLD (with SNPs C-statistic 0.81 (95% CI 0.75–0.88) vs. 0.77 (0.70–0.84) without SNPs; *p* = 0.047). Among all, the *PNPLA3*, *TM6SF2*, and *GCKR* allele variants associated with gene–adiposity interactions have an important role in NAFLD development and progression [47]. However, the only genetic predisposition does not account for the diversity in the risk of the onset and progression of the disease, hence the idea that NAFLD might be a multifactorial disease in which there may be an interplay between different predisposing factors. In this regard, an important role could be played by epigenetic modifications; thanks to which, changes in DNA expression can be achieved as a result of exposition to environmental factors without altering the linear DNA primary sequence [48].

Circulating microRNAs (miRNAs) are emerging as new biomarkers of MAFLD [49]. MiRNAs are small (18–25 nucleotides) non-coding RNAs that regulate post-transcriptional gene expression. Their binding to target messenger RNAs (mRNAs) inhibits translation from the nucleus to the cytoplasm of the codifying sequencing of the target genes [50,51]. Different miRNAs have the potential to bind complementary sequences in multiple mRNAs, influencing several pathways. Among them, miRNA-122 is the best studied in mice and adult models of MAFLD [52]. It reduces hepatic lipid production and increases fat oxidation in normal livers through different mechanisms. In fact, it blocks the expression of lipogenic enzymes and transcription factors such as Sterol Regulator Element-Binding Protein-1c (SREBP-1c), fatty acid synthase (FASN), 3-hydroxy-3-methylglutaryl coenzyme A (HMG-CoA) reductase, and two enzymes required for TG synthesis, 1-acyl-sn-glycerol-3-phosphase acyltransferase alpha 1 (AGPAT1) and diacylglycerol-acyltransferase 1 (DGAT1) [53]. Studies conducted on mice and adult subjects have shown that the miRNA-122 levels decrease parallel the advancement of hepatocellular damage; therefore, they might be used as a marker of NAFLD. Although there are conflicting data on the pediatric population [54], strategies to maintain the miR-122 abundance within the liver are currently being explored for HCC treatment [55,56]. MiR-192 is another increased miRNA in adults with NAFLD. It is highly expressed in the liver and in the serum, appearing as a good biomarker of the disease. Different authors have shown a six-times higher increase in adult patients with NAFLD [57,58,59,60]. It regulates the activity of Stearoyl-CoA desaturase 1 (SCD1), a lipogenic target. Therefore, a diet rich in fat reduces the miR-192 hepatic content to promote hepatic lipid deposition [59,61,62]. Moreover, the excessive introduction of fat foods seems to favor miR-192 export with the consequent activation of proinflammatory cells [60]. However, its use is limited, because it is not specific for liver. It is largely produced in the gut, and variations in the serum concentration may be influenced by several conditions involving this organ [63]. 

Given the close association existing between NAFLD and metabolic dysregulation, scientific interest has been focused on finding miRNA able to regulate liver lipogenesis, gluconeogenesis, and fat oxidation, with promising results. Among them, miR-155 has been one of the most studied. It represses SREBP-1c and FASN in hepatocytes interacting with the liver X receptor (LXR), thus reducing TG production. Data on adult patients with NAFLD have reported low serum and liver contents of this mediator. However, although several mi-RNA (mi-RNA30a, miR-27a and -27b, miR-26, and others) are emerging in mice studies in association with NAFLD, further studies on humans are necessary to bring to light their real role in the intricate pathogenesis of NAFLD and, above all, the potential diagnostic use for NAFLD.

## 5. NAFLD and Metabolic Dysregulation

NAFLD is often part of a complex clinical picture characterized by obesity, IR, and other metabolic alterations describing MetS [64,65,66,67]. As previously mentioned, to emphasize the tight connection between hepatic steatosis and metabolic disorders, the term NAFLD has recently been replaced by the creation of the term MAFLD, which stands for fatty liver presenting with other items of MetS [9,10]. Therefore, the complete comprehension of predisposing factors to MAFLD development should start from the knowledge of the numerous underlying medical conditions frequently associated.

### 5.1. Obesity and Diet

Obesity is the main risk factor related to MAFLD onset. The later appearance of obesity during adolescence increases the possibility of persistence of this condition in adulthood and, consequently, the occurrence of its associated complications [68,69]. The most remarkable event increasing MAFLD risk over lifetime is an early weight gain. In this regard, a longitudinal study conducted in Denmark showed that a weight gain among young people in the group ranging from 7 to 13 years is correlated with a higher NAFLD incidence as an adult [70]. However, the authors did not find a relationship between the starting value of the BMI and the risk of developing NAFLD later in life, suggesting a direct contribution of bodyweight gain in fat liver accumulation and the following events leading to NAFLD progression. Comparing the risk of developing cirrhosis in adulthood, an increased risk of around 16% per one-unit gain in the BMI z-score has been observed amongst children aged 13 years who presented a weight gain in the age range between 7 and 13 years [70]. Nevertheless, the degree of obesity does not always correlate with the severity of NAFLD in the pediatric population, suggesting that a combination of several factors is involved in determining the risk of NAFLD development [71,72]. Not surprisingly, the BMI is not considered an accurate parameter for the assessment of the obesity degree, whilst the waist circumference better correlates to the visceral fat accumulation. In this regard, the Bogalusa Heart Study showed a higher cardiometabolic risk and prevalence of MetS among normal weight centrally obese children (WHtR ≥ 0.5) compared to overweight or obese children without abdominal obesity (WHtR < 0.5) [73].

The pathogenetic connection between obesity and NAFLD might lie in the strict relationship existing between the fat storage capacity of adipose tissue and secondary hepatic involvement. Physiologically, the adipose tissue plays a fundamental role in obese subjects, because it removes the excess of lipids from the blood system, reducing their afflux to the liver. However, the achievement of the saturation fat threshold for adipose tissue results in adipose damage and inflammation with the production and release of proinflammatory cytokines and adipokines other than reactive oxygen species. In addition, it increases the afflux of FFAs to the liver, thus resulting in an increased fat liver content [74].

The circulating proinflammatory cytokines cause endoplasmic reticulum and mitochondrial stress responsible for hepatocellular involvement and predisposition toward the progression of NASH [75]. Moreover, the combination of IR and an impaired fat liver profile interferes with the balance existing within circulating FFAs, DNL, and hepatic TG clearance (mediated by both *β*-oxidation and elimination as VLDL). In fact, the secretion of hepatic VLDL into the bloodstream is reduced in subjects with NAFLD. Consequently, the excess of DNL and VLDL storage conduct leads to fat accumulation [76]. Nonetheless, a significative contribution to lipid disorders comes from environmental factors such as dietary habits, physical activity, and socioeconomic factors. Particularly, the daily physical activity levels have been described as inversely proportionate to the appearance of NAFLD [77]. Moreover, a high frequency of NAFLD typically distinguishes populations where a Western diet style predominates with a major consumption of processed products and/or greasy, salty, sugary, or poor in fiber foods [78,79,80]. 

Compared to adults, children prefer to consume ultra-processed foods, implying poor metabolic health [79]. Hypercaloric diets enriched in fat and fructose/sucrose may contribute to dangerous hepatic FA accumulation with a dual mechanism: by favoring the IR or by causing an increase of central adiposity, which are independent risk factors for MetS [81]. Saturated fats promote FA oxidation through peroxisome proliferator-activated receptor alpha (PPARα) and DNL in the liver [82]. Additionally, a reduced omega-3/omega-6 ratio has also been associated with an increased risk of NAFLD in children [83]. Therefore, it is clear about how not only the amount but, also, the quality of fat ingested contribute to hepatic steatosis. 

Recently, a linkage between the increased intake of added sweeteners and MetS has been observed. Certainly, obese children with NAFLD tends to consume a higher amount of carbohydrates than those obese without NAFLD. The high intake of carbohydrates positively influences the blood glucose levels, which are accountable for activating different intracellular pathways. Particularly, it has been reported that high blood glucose levels activate Carbohydrate Response Element-Binding Protein (ChREBP), a key regulator of insulin-independent glycolysis and DNL [84]. 

Special attention has been paid to the fructose intake and the increased risk of NAFLD development. Fructose is a highly lipogenic sugar naturally present in fruits and vegetables with a high fructuous content (e.g., artichokes, wheat, leeks, and garlic) and honey. Fructose, sucrose, and high-fructose corn syrup are largely used as added sweeteners. The fructose metabolism does not require insulin action, because it uses the transporter GLUT5 to move into hepatic cells, instead of GLUT1 and GLUT4-mediated internalization. Fructose is turned into fructose 6-phosphate by fructokinase inside the cytoplasm of hepatocytes and then hydrolyzed into fructose 1-6 bisphosphate thanks to fructose aldolase activity. The metabolite thus obtained enters the glycolytic/glucogenic pathways. In this way, ingested fructose alters mainly carbohydrates compared to the lipid metabolism. In fact, increases in IR, fasting glucose, and insulin levels have been correlated with fructose ingestion with a mechanism currently unknown [78]. However, postprandial lipedema is higher after fructose consumption. The reason lies in the insulin-independent induction of many hepatic lipogenic enzymes (e.g., pyruvate kinase, NADP+-dependent malate dehydrogenase, citrate lyase, ACC, FASN, and pyruvate dehydrogenase) and an increase in VLDL production and hepatic fat storage [85,86]. Additionally, extra fructose activates both ChREBP and SREBP-1c [87]. Finally, the fructose intake may favorite hepatocyte apoptosis, with hepatic fibrosis determining the increased prevalence of NASH [88]. All these findings underline that a healthy diet and the intake of unsaturated fats play a pivotal role in reducing the onset and progression of NAFLD. Hence, a reduction of dietary sugars is associated with the reduction of hepatic damage. Two different studies have observed that a reduction of added sugars and fructose to 10% and 4% of the daily energy intake for nine days in obese adolescents with a diet of the predominant sugar content conducts a decrease of the liver fat content, DNL, and fasting insulin [86,89]. Similarly, Schwimmer et al. explored the role of dietary sugars by performing a randomized study on a cohort of boys aged 11–16 years old with NAFLD and at least 10% hepatic fat content. In the group of patients on a low-sugar diet (less than 3% of their daily energy from added sugars), the authors showed a reduction of the hepatic fat content by about 8% versus only 1% change in the control group (with a usual diet), as well as a drop in the levels of transaminases. No difference was observed in the fasting insulin or TGs [90]. Therefore, a dietary sugar reduction can be a first-line treatment in obese adolescents with NAFLD. 

### 5.2. Insulin Resistance and T2DM

Different cohort studies conducted on large populations of adults [91,92] and young people [93,94] have shown the coexistence of IR and NAFLD. It is not yet clear the kind of relationship that exists between these two conditions, but further studies are in progress to evaluate the possible role of IR both as a causal risk and as a consequence of NAFLD hepatic damage. The pathogenetic mechanisms linking NAFLD and MetS are complex and still not completely explored. Nevertheless, it was observed that the persistence of IR in youth is associated with an increased Hepatic Fat Fraction (HFF), while the absence of fat hepatic accumulation improves the insulin sensitivity and glucose metabolism homeostasis. Hence, it has been supposed as a pivotal role of IR to contribute to systemic metabolic alterations [95]. 

Physiologically, insulin is an anabolic pancreatic hormone acting on three target organs: in the muscles, it promotes the uptake of circulating glucose; in the liver, it mediates its hypoglycemic action by inhibiting gluconeogenesis and stimulating the uptake of peripheral glucose; and in adipose tissue, it inhibits lipolysis and promotes FA storage in the adipocytes in the form of TGs [96]. In this way, it reduces the production of FFAs, which could be turned into glucose in the liver when it exceeded the hepatic saturation threshold for fat [97]. The scientific proof of this linkage between Hepatic Glucose Production (HGP) and IR was well-explained by Caprio et al., who measured these two parameters in obese insulin-resistant adolescents during a hyperinsulinemic–euglycemic clamp [98]. Carrying out a weight-matched comparative assessment between obese adolescents with altered insulin sensitivity and obese insulin-sensitive control adolescents, the former exhibited fasting hyperglycemia and hyperinsulinemia, consequently associated with increased rates of HGP, impaired insulin-mediated suppression of lipolysis, and impaired insulin-mediated suppression of HGP [98].

Not surprisingly, nearly 30% of children with MAFLD also have T2DM or prediabetes. In particular, children with T2DM have greater odds of developing NASH (43.2%) compared with prediabetes (34.2%) or those with normal glucose values (22%) exposing them to a greater long-term risk for adverse hepatic outcomes [99]. The strict link between T2DM and NAFLD is being increasingly acknowledged in recent years. T2DM is not only an independent risk factor for NAFLD, but conversely, NAFLD interferes with glucose metabolism, increasing the risk of developing T2DM [100]. In this regard, Armstrong et al. [101] reported a doubled risk of developing NAFLD in diabetic compared to non-diabetic patients. Simultaneously, hepatic steatosis increases the risk of T2DM by two to five times.

However, on the other hand, it has been assumed that the NAFLD condition could play a role in IR development. D’Adamo et al. examined the contribute of hepatic steatosis (value through MRI) in inducing IR in the liver, adipose tissue, and muscles. The authors showed that IR in the liver is associated with an impaired insulin sensitivity also in adipose tissue and muscles. Therefore, it could be inferred that ectopic fat accumulation might be the starting event to systemic IR development [102]. Moreover, a recent Mendelian Randomized (MR) study evaluated the early causal role of the NAFLD-related genetic risk score (GRS) in determining the changes in the Homeostatic Model Assessment for Insulin Resistance (HOMA-IR) values, a fasting index of IR [103]. The analysis was carried out in two cohorts of adult and pediatric patients with genetically influenced-NAFLD and did not confirm a causal link between these two conditions, suggesting a possible role of NAFLD as a bystander. However, those results contrast previous evidence about the increased risk of diabetes in patients with NAFLD [104,105]. A plausible hypothesis is that the most severe form of liver damage could interfere with the balance of carbohydrates, independently from fatty liver accumulation [106,107]. Furthermore, a specifical predisposition toward the development of diabetes related to a specific polymorphism or to alternative pathogenetic pathways capable of reducing the systemic insulin sensibility cannot be excluded [103]. However, changes in the body fat distribution could also play a potential role in determining the IR. The first evidence derives from a positive correlation between both the high intramyocellular lipid content (IMCL) and visceral adiposity (VAS) with impaired glucose tolerance (IGT) in obese adolescents [108]. Later, this data was confirmed by a wide multiethnic cohort of obese adolescents in which it has been shown that the insulin sensitivity is inversely correlated with VAS. Instead, subcutaneous fat accumulation seems to not alter the peripheral response to insulin [98]. It is not understood if the degree of hepatic steatosis influences the severity of IR. However, a longitudinal study conducted by Kim et al. on a cohort of multiethnic obese adolescent evaluated if the baseline HFF value through the MRI technique could influence the IR development. They showed that a high baseline HFF in adolescents is associated with a persistence of IR during the lifetime, supposing a direct contribution of fat liver in the changes in insulin sensitivity [109].

TGs are the main form of hepatic fat in patients with MAFLD and derive from the esterification of a molecule of glycerol with three FA chains. They are not hepatotoxic in contrast to FFAs, which are able to modify the oxidative state of a hepatic cell and, therefore, trigger the hepatocellular integrity [110]. Additionally, the increased availability of circulating FFAs reinforces peripheral and hepatic IR, perpetuating and exacerbating fat-induced metabolic damage [111]. The establishment of a condition of systemic IR alters not only the glucose homeostasis but, by reducing peripheral glucose uptake and promoting lipolysis, increases the hepatic afflux of substrates for gluconeogenesis. 

### 5.3. Alterations in Lipid Metabolism

NAFLD is often associated with the atherogenic lipid profile [112,113]. In adult patients with NAFLD, it has been assessed a higher frequency of abnormalities in circulating lipids characterized by increased levels of the serum total cholesterol, low-density lipoprotein cholesterol (LDL-C), and VLDL-C levels associated with lower rates of high-density lipoprotein cholesterol (HDL-C) than heathy subjects [114,115]. Thereafter, this association has also been confirmed in the pediatric population. Nobili et al. observed a more atherogenic profile in children with NAFLD as the severity of the hepatic injury increases the TG/HDL-C, total cholesterol/HDL-C, and LDL/HDL-C ratios [116]. Subsequent studies have confirmed this association, suggesting the TG/HDL-C ratio as a cardiovascular risk marker in pediatric patients with NAFLD and the TG levels as an indicator of NAFLD severity. However, an altered lipid profile often characterizes obese youth [117]. The current epidemiological data report the highest rate of total serum cholesterol in American children aged 16–19 years (8.9%). Therefore, a primary intervention of circulating lipids in determining hepatic fat accumulation cannot be excluded [118].

### 5.4. Prenatal Factors

Although mounting evidence highlights the relation between hepatic steatosis, obesity, and IR, as the starting point towards MAFLD progression [8], obesity is not a constant element of pediatric NAFLD. In fact, non-obese children who have developed NAFLD at an early age have been described in the literature, ranging from 1.5% with an ultrasound-based diagnosis to 5% in autopsy evaluations [70]. In this regard, a further possible scientific explanation of the pathogenetic mechanisms behind MAFLD evolution is an exposition of different prenatal and childhood factors of individuals genetically predisposed toward early onset of the disease independently from the BMI values.

**Figure 3:**
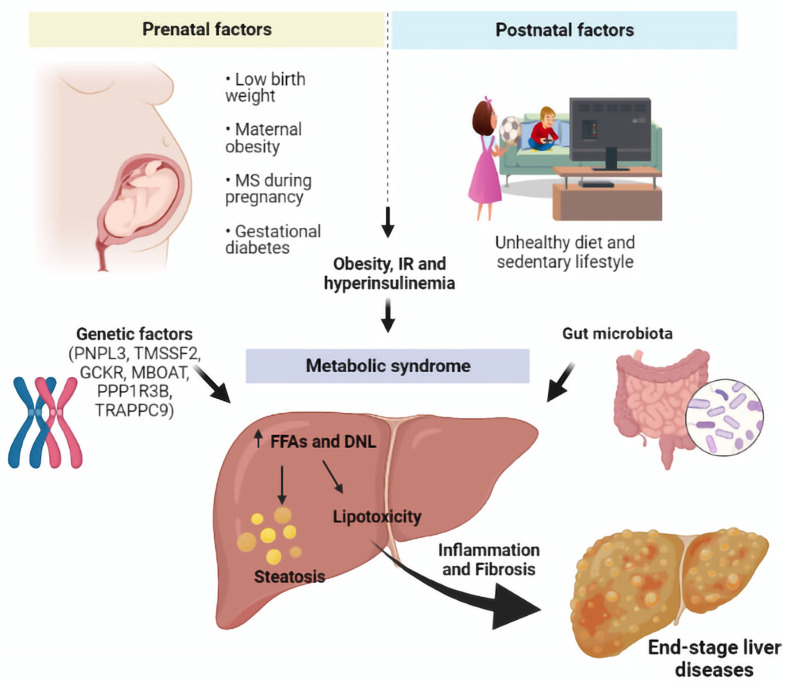
The role of prenatal and postnatal factors in MAFLD.

Over the years, it has emerged that several maternal and offspring features might influence the appearance of NAFLD. Among them all, a low birth weight, maternal obesity, metabolic syndrome during pregnancy, and gestational diabetes seem to be correlated with the offspring’s hepatic disease [119,120] (Figure 3). 

## 6. The Role of Microbiota

Over the last years, much progress has been done in establishing the role of gut microbiota in many disorders, especially metabolic diseases [121]. The hypothesis that microbiota could cause a worsening of the disease should be considered not only for better knowledge of the underlying pathophysiology but also as it could represent a potential target of treatment. Indeed, gut dysbiosis seems to be involved not only in the development of NAFLD but is also responsible for its progression to NASH and, eventually, cirrhosis, as well as hepatocarcinogenesis. As a result, the gut microbiota is currently emerging as a noninvasive biomarker for the diagnosis of the disease and for the assessment of its severity [122]. On the other hand, there is an intricate network of cross-talking between the gut, microbiome, and liver through the portal circulation, creating a mutual relationship in which they are able to influence each other. In this new model of a “gut–liver axis”, liver diseases can also alter the gut microbiota [123].

As for the pathophysiology, intestinal microbiota can contribute to the development of hepatic steatosis through multiple mechanisms, including an increased dietary energy harvest, the regulation of choline metabolism, the production of short-chain fatty acids (SCFAs), and the modulation of bile acid signaling. More importantly, the hypothesis of a “leaky gut” has emerged, according to which intestinal dysbiosis altered the gut endothelial barrier function, allowing microbes and/or microbial products (endotoxins, lipopolysaccharide (LPS), and peptidoglycan) to enter the portal circulation. Translocated bacteria or their products promote the activation of the inflammatory cascade and the production of inflammatory cytokines, causing liver inflammation and fibrosis. However, it is not clear whether an increased intestinal permeability should be considered as a cause or, rather, a consequence of NAFLD [122,124].

Several scientific works have been published to illustrate the strict relationship existing between microbiota and metabolic disease, i.e., obesity [125,126,127] and T2DM [128]. Moreover, an interesting case–control study reported that a poor variability of the microbiota population at the age of 6 months is associated with an increased risk of obesity by the age of 7 years compared to the controls [129]. Moreover, breast-fed infants have a lower risk of developing NAFLD later in life, probably because breastfeeding influences the microbiota composition, providing oligosaccharides as prebiotics [130]. Considering that obesity, IR, and NAFLD/NASH are clinically associated with each other, the hypothesis that the microbiota is involved in NAFLD development cannot be excluded.

The gut microbiota represents a source of TLR ligands. Any change in their settlement acts as a potential trigger in the activation of TLR signaling in the liver, which can induce inflammation under certain conditions. Thus, many researchers are trying to identify the specific bacteria changes linked to the development of NAFLD [124]. In this regard, studies have shown that the levels of *Firmicutes* are increased, whereas those of *Bacteroidetes* are decreased in obesity and its related diseases, suggesting that an increased *Firmicutes/Bacteroidetes* ratio could prepare for the development of obesity [131,132]. Moreover, Boursier et al. demonstrated that NAFLD severity was associated with gut microbiome alterations and shifts in the metabolic function of the microbiome. More specifically, they found that *Ruminococcus* bacteria were independently associated with fibrosis [133].

Recent data in adults have also shown that fatty liver disease can develop in individuals with a normal BMI. NAFLD; in lean individuals with a BMI < 25 kg/m^2^, this is defined as hepatic steatosis. This new entity occurs in metabolically obese patients with or without a coexisting increased waist circumference and visceral adipose tissue. In this subset of patients, the gut microbiota seems to play an interesting role within the pathophysiological process behind. Particularly, there is evidence of a distinct gut microbiota profile compared with the healthy controls rich in the species implicated in the generation of liver fat, such as *Dorea*, which is involved in the pathogenesis and progression of NASH. On the other hand, a decrease of several species accounted for as protective against NAFLD, such as the *Marvinbryantia* and *Christensellenaceae R7* groups, has been documented [134]. According to the data extracted from NHANES, hepatic steatosis was documented in 7% of lean adults with ultrasound evidence [135]. On the other hand, pediatric studies evaluating the prevalence of lean NAFLD are very limited. Interestingly, a recent study conducted in the US during 2005–2014 showed that the mean estimated prevalence of suspected NAFLD among lean adolescents was 8%, pointing out the presence of metabolic disorders such as low HDL, hypertriglyceridemia, and IR in this population [136].

Despite this great variety of evidence, a consistent microbiota signature characterizing individuals at different stages of the disease does not exist. Probably, demographic characteristics (such as age, sex, and ethnicity) but, also, the type of histological damage or comorbidities (including obesity and T2DM) could interfere with the microbiota composition and, consequently, MAFLD development. Once this strict connection is assumed, the microbiome represents a potential noninvasive marker of disease severity in NAFLD that could help to determine the risk of disease progression toward NASH and more severe fibrosis or, eventually, HCC [122]. Moreover, it could become a promising therapeutic target for NAFLD, whose treatment options still remain limited to date [122].

## 7. Pathogenesis

Despite the majority of discoveries related to pathogenetic mechanisms derived from NAFLD, the new terminology MAFLD shares similar driving factors with NAFLD onset and evolution [137]. In this regard, a first proposed pathogenetic model was the “two-hit hypothesis”, in which two different events act separately at different times, causing hepatic damage [138]. The “first hit” is directly related to bad eating habits, a sedentary lifestyle, and mutations in multiple genes involved in glucose and fat metabolism, causing metabolic dysregulation and, subsequently, fat accumulation. Later, the presence of an inflammatory setting may promote necroinflammation and fibrosis, leading to end-stage liver disease [139,140]. However, considering the complex interplay between different drivers in the cascade of events bringing about hepatic steatosis, inflammation, and fibrosis, a novel theory named “multiple-hits theory” has been proposed [141,142]. According to this model, multiple synergistic events proceed in parallel conduct to liver inflammation, which, in certain instances, may also precede steatosis. However, in an attempt to establish a temporal link between the main pathogenic events, the first step could be represented by fatty acid (FA) accumulation in hepatocytes promoted by an excessive dietary intake of carbohydrates and lipids [139,140].

Certainly, IR represents an important condition in the regulation of these metabolic pathways. Particularly, under certain circumstances, the insulin-mediated inhibition of lipolysis is thought to be impaired, with a consequent rise of the inflow of FAs to the liver [143].

Moreover, hyperinsulinemia, together with some proinflammatory cytokines locally produced, acts on the liver, regulating the expression of SREBP-1c, an important factor that activates the lipogenic genes [144]. Simultaneously, hyperglycemia stimulates ChREBP, promoting the expression of more lipogenic genes [145]. 

Thus, the combined action of SREBP-1c and ChREBP activates the enzymes necessary for the conversion of excess glucose into FAs, a process defined as DNL. The consequent altered lipid metabolism in the liver causes the transformation of FAs into free FAs (FFAs); these molecules can both be oxidized in the mitochondria to form ATP or esterified to produce TG, which are stored in the liver or incorporated into the VLDL for secretion [139,140]. Within this cycle, acetyl-CoA carboxylase (ACC) is a fundamental enzyme that catalyzes the rate-limiting step of acetyl coenzyme A (CoA) to malonyl CoA conversion and modulates mitochondrial FA oxidation. Therefore, it represents a potential therapeutic target in order to treat the dysregulation of hepatic FA metabolism [146]. In addition, AMP-activated protein kinase (AMPK) and malonyl CoA appear to be involved in energy balance regulation. Malonyl CoA is an allosteric inhibitor of carnitine palmitoyltransferase (CPT-1), the enzyme that controls the transfer of long-chain fatty acyl (LCFA) from the cytosol to the mitochondria, where they are oxidized. Therefore, when the malonyl CoA levels are elevated, CPT1 is inhibited, and the esterification of LCFA to form TG and diacylglycerol (DAG) is favored [147,148]. On the other hand, fuel deprivation and increased energy expenditure are able to promote the activation of AMPK and the decrease of the malonyl CoA levels in peripheral tissues, reducing the accumulation of TGs in adipocytes and other cells [148]. Interestingly, studies conducted on mice treated with AMPK activator (AICAR) have observed a reduction of the ectopic lipid deposition, showing a decreased TG content in hepatic and muscular tissues [149]. Similar results also came from studies carried out on mice undergoing treatment with empagliflozin, which has been shown to improve hepatic steatosis through the activation of AMPK signaling [150]. When FFAs are over-accumulated or their disposal is not timely, the redundant FFAs act as substrates to produce lipotoxic lipids (such as oxidized phospholipids), causing hepatocyte metabolic stress and damage or death [151].

The consequent development of ‘lipotoxicity’ is one of the main triggers of inflammation and cell death or autophagy, causing steatohepatitis. More specifically, the intracellular lipid excess in the liver leads to mitochondrial dysfunction, endoplasmic reticulum stress (ERS), oxidative stress, the perturbation of intracellular signaling pathways, and the release of danger-associated molecular patterns (DAMPs) [152]. The expression of ERS-related proteins such as the transcription factor C/EBP homologous protein (CHOP) causes the activation of death receptor DR5. Moreover, binding the c-Jun N-terminal kinase (JNK), the palmitate-induced CHOP/c-Jun complex promotes the expression of PUMA, a proapoptotic BH3-only protein, enhancing its apoptotic effect. These modulators ultimately contribute to mitochondrial dysfunction and caspase cleavage, inducing cell death during hepatocyte lipotoxicity [153]. Caspases belong to a family of cysteine proteases that play a key role in the progression of NAFLD/NASH, since they are able to control liver apoptosis and inflammation [154]. Interestingly, Ferreira et al. showed that caspase-3 and -2 activation increases in the liver during disease progression from simple steatosis to severe MAFLD. More importantly, they found that JNK phosphorylation was significantly increased in patients with NASH compared to simple steatosis in both muscles and the liver, suggesting an additional mechanism of connection between apoptosis and IR at different NAFLD stages [155].

In addition, the increased release of proinflammatory cytokines (interleukin-6 (IL-6), tumor necrosis factor (TNF) α, and C-Reactive Protein (CPR)) and decreased release of adiponectin are associated with IR presented at both the hepatic and systemic levels [156]. Particularly, the innate immune response mainly regulates aseptic inflammation triggered by metabolic stress in the liver promoting the release of IL-1β and IL-18 and C-C chemokine ligand type 2 and type 5 (CCL2 and CCL5), together with C-C chemokine receptor type 2 and type 5 (CCR2 and CCR5), which, in turn, are able to induce hepatic cell injury and liver fibrosis by increasing immune cell aggregation and infiltration and amplifying the inflammatory response [157,158].

Recently, the third hit of the “multiple-hits” theory has emerged, referring to the effects of repairing mechanism activation following cellular damage due to the previously described processes. As a result of repetitive liver injury, dysregulated hepatocytes or inflammatory cells elicit paracrine signaling, which promotes hepatic stellate cell (HSC) activation, which is also mediated by circulating factors (e.g., adipokine and FA) released by visceral adipose tissue or the intestinal microbiome [159]. Particularly, reactive oxygen species (ROS) derived from the endoplasmic reticulum and NADPH oxidase (NOX) in apoptotic hepatocytes take part in HSC activation [159,160,161]. Moreover, since the receptors for advanced glycation end products (RAGEs) are highly expressed in HSCs, the diet can also have an influence on liver fibrotic evolution. In fact, ROSs are also generated in AGE formation, and oxidized RAGE stimulates NOX1, which contributes to ROS production in HSCs [161], causing further damage. Additionally, pathogen-associated molecular patterns (PAMPs) and DAMPs, as well as endotoxins, derived from the intestinal flora could directly promote fibrosis by signaling through innate immune receptors like TLR4 expressed on HSCs [159], suggesting that the activation of the innate immune systems, including TLR signaling, represents a pivotal event in chronic liver disease [162]. In this regard, one of the most effective fibrogenic cytokines is transforming growth factor β (TGF-β), able to trigger the HSCs that produce type I collagen through a signaling pathway involving Smad proteins, followed by platelet-derived growth factor (PDGF), another pro-fibrogenic cytokine, which encourages the proliferation and migration of HSCs [163,164,165]. In addition, hepatic macrophages can also polarize toward a proinflammatory phenotype, and their TLR4 signaling promotes the release of TGF-β in response to metabolic insults [166]. Therefore, under the stimulation of the abovementioned profibrotic factors, HSCs become the main effector cells in the process of hepatic fibrogenesis, stimulating the production of fibroblasts, portal vein fibroblasts, and myofibroblasts [167], which produce extracellular matrix components and proinflammatory mediators, contributing to the profibrogenic environment [163]. 

However, the overactivation of these healing processes leads to the onset of progressive liver fibrosis [90,91], especially in individuals with increased susceptibility to liver fibrosis due to gene polymorphisms, such as PNPLA3, TM6SF2, and HSD17B13 [159].

Recently, multiple novel pathways involved in liver fibrosis have emerged, offering the possibility to detect new therapeutic targets [168]. Among them, apoptosis signal-regulating kinase 1 (ASK1), a member of the mitogen-activated protein kinase (MAP3K) family, activates the downstream JNK 1/2-mitogen activated protein kinase 14 (p38) signaling cascade, triggering hepatic inflammation and fibrosis in response to metabolic stress signals during the development of MAFLD [137,169,170]. 

In conclusion, the immunopathogenesis of NAFLD can be synthetized into two different mechanisms: the first is the increased availability of fats derived from a diet responsible for the elevated level of FFAs circulating, increased DNL, and decreased release of hepatic TGs as VLDL. The second mechanism includes the activation of oxidative stress in which lipid peroxidation, mitochondrial dysfunction, and the release of inflammatory mediators can mediate secondary hepatic injury, leading to NAFLD progression [151,171]. 

## 8. Diagnosis and Outcomes

NAFLD is a diagnosis of exclusion based on the presence of hepatic steatosis and, especially, on the exclusion of other causes of hepatic steatosis besides NAFLD [172]; on the other hand, the new MAFLD definition highlights the coexistence of hepatic steatosis and metabolic dysfunctions, underlining the strict relation to disease etiology and pathogenesis [8]. 

Consequently, an international group of experts established MAFLD diagnostic criteria for pediatric patients that included histological, imaging (ultrasound), or blood biomarker (e.g., ALT) evidence of steatosis, in association with excess adiposity, the presence of prediabetes or T2DM, or evidence of metabolic dysregulation. The latter is defined by two or more altered results of the standardized biometric parameters for age and sex (including waist circumference, blood pressure, TGs, HDL-C levels, fasting glucose, and TG/HDL-C ratio), with a different cutoff for each ethnic group [10] (Table 1).

However, if the definition of MetS in adults is well-established, it is still a discussed matter in children. In fact, during childhood, cutoff points cannot be used to define abnormalities considering that the blood pressure, lipid levels, and anthropometric variables change with age and pubertal development; thus, mainly values above the 90th, 95th, or 97th percentiles for gender and age are systematically used. In children older than 16 years, the diagnosis of MetS is currently achieved according to the International Diabetes Federation (IDF) adult criteria. In the group of patients aged 10–16 years, a MetS diagnosis is made when there is evidence of abdominal obesity according to age- and gender-specific percentile curves of the waist circumference (≥90th percentile) in association with two or more of the following metabolic factors: hypertriglyceridemia, low HDL-C, high blood pressure, or glucose intolerance. Additionally, in the youngest children between 6 and 10 years old, MetS cannot be diagnosed, but in the presence of a waist circumference over or equal to the 90th percentile, further investigations should be made [173].

Compared with NAFLD, patients with MAFLD have shown higher levels of liver enzymes, blood lipids, BMI, waist circumference, and blood glucose with greater proportions of diseases at a high risk of a negative outcome [13,21]. Moreover, Yamamura et al. reported that advanced hepatic fibrosis is more associated with MAFLD patients than NAFLD [174], with significant implications in determining higher estimates for all-cause mortality among MAFLD patients compared to NAFLD [175]. Since fibrosis represents a main prognostic factor of MAFLD, and it is chiefly driven by metabolic inflammation, the traditional dichotomous classification into NASH versus non-NASH based on the evidence of hepatic ballooning should also be abandoned considering the significant sampling variability with which this histological evidence was submitted [176]. Moreover, MAFLD has increased the overall risk of all causes of mortality in a greater magnitude than NAFLD, independently of the known metabolic risk factors [12]. While adults with MAFLD have shown a 17% higher risk of all causes of mortality, NAFLD per se has not been associated with an increased risk of all-cause mortality after proper adjustment for the metabolic risk factors [175]. 

These first findings suggest that prognostic differences in the newly introduced diagnostic criteria for the two terms exist considering that MAFLD’s definition tends to exclude individuals with a lower mortality risk, preferably including subjects with a higher risk [12].

Currently, several studies are being conducted with the aim to compare the different outcomes in clinical cohorts in whom the NAFLD versus MAFLD diagnostic criteria are applied. These data will likely represent a significant landmark in consensus building efforts in the near future [176]. However, the proposed change of nomenclature as MAFLD does not allow to describe the entire spectrum of the disease [11]. In fact, the wide range of clinical phenotypes of NAFLD strongly related to its multifactorial etiology, pathophysiological heterogeneity, and genetic polymorphisms suggests the need to define different multiple subtypes of MAFLD [177]. In this regard, Singh et al. suggested a ‘MEGA-D’ classification of MAFLD, an acronym that summarizes its typical multiplicity by representing five subtypes of the disease: M–Metabolic syndrome, E—Environmental stressor, G—Genetic Factor, A—Bile Acid dysregulation, and D—gut Dysbiosis related to NAFLD [178]. Another physiopathogenetic element on which the scientific interest has been focusing is represented by the lipid profile. The idea is to identify various patterns of lipid metabolism to describe several phenotypes of NAFLD according to the different pathogenetic pathways involved into both ‘M-subtypes’ and ‘non-M subtypes’ [177,179]. Interestingly, Wu et al. conducted a cross-sectional study to characterize the lipid profiles associated with liver the fat content in MAFLD patients with different phenotypes [180]. The study reported different compounds of lipids and lipoproteins in MAFLD patients with T2DM or overweight/obesity than those who were lean/normal weight. Particularly, in the former, a positive correlation was found between fatty liver storage cholesterol, TG, (HDL-C), (LDL-C), apolipoprotein B, apolipoprotein E, and lipoprotein(a). A similar trend has been observed for TG in those with T2D and for HDL-C in patients who were lean/normal weight. From this evidence, a predictor model of MAFLD based on individual lipide profiles has been hypothesized [180]. 

Others subclassifications of NALFD have been obtained based on demographic factors (age, gender, and ethnicity) and clinical and laboratory findings, although this modality of subtyping the disease still needs to be approved clinically [181]. Many attempts have been made using a genotype profile to subclassify subjects with hepatic involvement. Hoang et al. [182] proposed score-based subtypes of NAFLD evaluating the gene-level NAFLD activity (NAS) and gene-level fibrosis stage (gFib) scores. Comparing them, a prediction of both the risk of progression of the disease and the response to therapy has been assessed [182]. 

## 9. Treatment

The lack of guidelines for the management of the disease is one of the most important issues influencing the outcome of pediatric patients affected with MAFLD. Considering the tight association existing between obesity and MAFLD, lifestyle interventions (dietary changes, behavioral modifications, and physical exercise) have been the only proposed treatment strategy for a long time. In this way, the main goal is to achieve a weight loss that can reduce the body fat content and induce changes in the metabolic profile. Particularly, it has been documented that a dietary sugar reduction, especially in fructose intake, is associated with an improvement of the hepatic steatosis in obese adolescents [183]. Similarly, a quality adjustment of fats ingested with n-3 PUFA [184,185] and/or docosahexaenoic acid supplementation [186] are considered a safe and efficacious tool for the treatment of NAFLD in children. However, this intervention plan failed to appear as successful over time. In fact, it has been proven that a weight loss of more than 7–10% is associated with a reduction in steatosis and inflammation in most of the patients affected with MAFLD. Unfortunately, the percentage of adult and pediatric patients able to maintain a healthy lifestyle for a long time is very low, with a rapid step back toward the starting weight [187,188,189,190,191]. Therefore, changes in lifestyle should be considered only the first step of treatment for such a complex disease. However, since dietary habits and lifestyle play a key role in the prevention and treatment of MAFLD, the implementation of feeding studies identifying an effective nutritional strategy able to reduce the risk of liver disease should be supported [192]. 

## 10. Pharmacological Treatment

**Figure 4:**
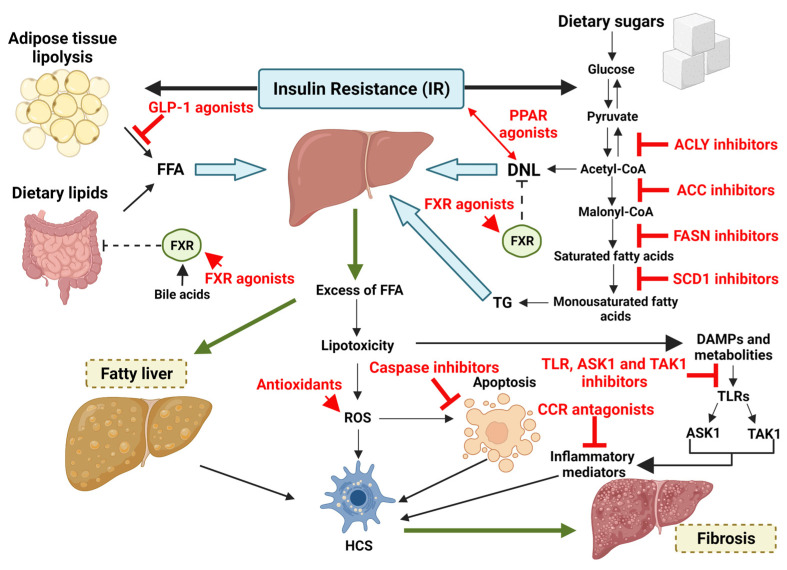
Possible therapeutic targets for the treatment of MAFLD and its subsequent fibrosis.

Many drugs are being studied for MAFLD/NASH treatment, especially in adults, without showing any current practice utility. Taking into account the strong contribution of metabolic dysregulation in NAFLD pathogenesis, the actual scientific research is focused on finding molecules capable of interfering with the intricate pathways of carbohydrates and lipidic metabolism and simultaneously able to stop the devasting cascade of inflammation and fibrosis [193]. Considering the numerous overlapping molecules that exist between MAFLD and NAFLD in terms of prevalence, risk factors, and pathological and metabolic traits, the actual knowledge about NAFLD should be used to obtain druggable targets for the treatment of MAFLD and its subsequent fibrosis [12,13,194,195] (Figure 4). However, so far, poor and not consistent results have been achieved in practical terms. 

Researchers are faced with trying to reduce the afflux of FA derived from adipose tissue lipolysis. In that direction, a potential role could be played by the glucagon-like peptide-1 (GLP-1) analog. GLP-1 is a peptide hormone produced and excreted by intestinal L cells, promoting insulin secretion and improving glucose homeostasis. Moreover, it reduces the liver non-esterified fatty acid (NEFA) overload caused by triglyceride decomposition. Beyond its metabolic effects, GLP-1 can delay gastric emptying and limit body weight increase, as well as it can inhibit inflammation and cell apoptosis [196]. For these reasons, GLP-1 receptor agonists have been proposed for the treatment of MAFLD. Liraglutide is the best-studied among all GLP1 agonists. In a phase 2 study (NCT01237119) on adult patients with NAFLD treated with Liraglutide, besides a reduction in body weight, a histological improvement with a reduction of the fibrosis index was shown [197]. Nevertheless, exenatide, another GLP-1 receptor agonist, proved to have a greater efficacy in improving the fibrosis stage than liraglutide [198]. Starting from this evidence, many studies have been initiated using different molecules belonging to this pharmacological class (e.g., Semaglutinde, Cotadutide, and Tirzepatide) [199,200,201]. However, none of them are yet recommended to treat patients with NAFLD/MAFLD. 

Moreover, taking into account that a part of the circulating fats derives from foods ingested, efforts have been made to identify druggable pathways. For this purpose, a possible therapeutical use of Fanitol X Receptor (FXR) agonists has emerged. FXR is a nuclear receptor expressed in the liver and small intestinal mucosa. It mediates a negative regulation on intestinal lipid absorption using bile acids as signals of excess circulating fats. Particularly, FXR activation brings a downregulation of the expression of key lipogenic genes in the liver and reduces the hepatic lipid levels blocking DNL and promoting fatty acid oxidation [202]. In addition, it reduces the rate of IR in muscle and adipose tissue. Together, these effects conduct a reduction of lipotoxic lipids that, if in excess, can lead to a cascade of events ending in hepatic fibrosis [203]. The idea to use FXR agonists to treat patients with NAFLD derives from rodent models in which it has been proven that the deletion of FXR in the liver is followed by the appearance of liver steatosis, inflammation, and fibrosis [204]. Obeticholic acid (OCA) is the first FXR agonist that synthetizes as a competitive ligand of FXR. In fact, it binds to the receptor with 100-fold more potent affinity than the endogenous ligand chenodeoxycholic acid [204]. Promising results derive from both animal and clinical trials. The former have shown that OCA reduces the fatty liver accumulation, liver damage progression, and it simultaneously brings an improvement of the metabolic state [204,205]. Instead, clinical trials involving patients with advanced histological states (NASH, cirrhosis, and fibrosis) have observed a marked improvement of inflammation and fibrosis [206,207]. However, two adverse effects have emerged consequently for OCA use: a mild-to-moderate dose-dependent pruritus and an increase of the LDL-C levels during treatment that raises the risk of atherosclerosis in NASH patients with an already impaired metabolic profile [207]. In this regard, a CONTROL phase 2 study (NCT02633956) was initiated with the aim to evaluate the potentially favorable effect of combination therapy OCA–statin. The results derived after 16 weeks of therapy reported a slight reduction of the LDL-c levels in NASH patients, with a good tolerance and safety [208]. On the back of these data, further molecules have been identified as FXR agonists (Cilofexor, EDP-305, Tropifexor (LJN452) (NCT03517540 and NCT04065841), Nidufexor (LMB763), PX-104, EYP001, and TERN-101), for whom further studies are necessary to evaluate their efficiency and safety profiles [209,210,211,212].

In an attempt to stop the hepatic accumulation of TG, the possibility of reducing DNL by blocking a key enzyme in this process has been suggested. Among all the therapeutic targets studied, ACC is one of the most popular, being able to regulate the oxidation of mitochondrial fatty acids through the malonyl-CoA levels. Particularly, Firsocostat (or GS-0976 and NDI-010976) is the name of a molecule synthetized to inhibit ACC in a dose-dependent manner that has shown promising results in adult patients with NASH in terms of the reduction of hepatic steatosis and fibrosis [213,214]. However, most patients treated with Firsocostat experience a remarkable increase in the serum TG levels, limiting its application. Probably, the decreased polyunsaturated fatty acid production starting from malonyl-CoA promotes the expression of SREBP-1, causing increased VLDL secretion and peripheral TG accumulation [146]. Another therapeutic target could be represented by ATP-citrate lyase (ACLY), a lipogenic enzyme responsible for the synthesis of precursors of FA and cholesterol, such as cytoplasmic oxaloacetate and acetyl-CoA [215]. Additionally, since it is directly involved in the production of proinflammatory factors and in fibrogenesis, high levels of ACLY have been documented in livers of patients with NAFLD [215,216,217,218]. Using this evidence, it has been suggested to use ACLY inhibitors to reduce hepatic steatosis, oxidative stress, and the production of inflammatory mediators, contributing to the improvement of fibrosis in metabolic-induced liver disease [204,219,220]. Interestingly, another potential therapeutical target is represented by FASN, a modulator of hepatic DNL that catalyzes the synthesis of palmitate from acetyl-CoA and malonyl-CoA [221,222]. A phase 2a study (FASCINATE-1 (NCT03938246)) recently attested to a beneficial effect of TVB-2640, a FASN inhibitor, in the treatment of the advanced stage of NAFLD/MAFLD [223]. Therefore, FASCINATE-2 (NCT04906421) has been launched to evaluate the long-term effects of a 52-week therapy [224]. A different therapeutic target in the context of DNL is represented by SCD1 that synthetizes unsaturated FA, promoting NAFLD evolution towards NASH and fibrosis. Arachidyl-amidocholanoic acid (Aramchol) downregulates SCD1 and, thus, inhibits DNL in the liver, reducing steatosis and inflammation and improving fibrosis in mice [225]. In fact, many studies have documented its overexpression in activated HSCs, where it stimulates Wnt signaling [226,227].

Novel therapeutic strategies are basing their intervention on the attempt to block lipid oxidation and the consequent hepatocellular damage derived from lipotoxicity. In this regard, scientific attention has been focused on the role of PPARs such as PPARα, PPARγ, and PPARδ, considering their central role in the regulation of metabolic homeostasis and inflammatory response in the liver [204,228,229,230]. Based on this evidence, it has been proposed to use PPAR agonists (e.g., Pirfenidone, Elafibranor, and Saroglitazar) in the treatment of liver fibrosis, exploiting their anti-steatogenic and antifibrotic effects [231,232,233]. Another natural antioxidant that has shown promising results for the treatment of advanced stages of MAFLD is represented by vitamin E. In fact, it prevents plasma lipid and LDL peroxidation and maintains the structural integrity of cells, protecting them from damage caused by lipid peroxidation and ROS. In this regard, vitamin E supplementation could mediate its antioxidative effects, improving the histological pattern of adult nondiabetic patients affected with NASH [234,235]. Recently, a possible role in the MAFLD therapeutic landscape has also been suggested for vitamin D supplementation. Although vitamin D deficiency is widespread among the general population, several studies have observed that low levels of vitamin D are associated with an increased risk of developing steatosis, necroinflammation, and fibrosis both in children and adults [236,237]. Therefore, it could be reasonable to carry out vitamin D supplementation in all children with biopsy-proven NAFLD with the addition, if applicable, of docosahexaenoic acid supplementation [238]. 

Since an increased rate of hepatocyte apoptosis due to hepatic fat accumulation and inflammation has been documented, caspase inhibitors have also been proposed as potential therapeutical agents [154,239,240,241,242]. Among them, Emricasan (IDN-6556) has been the first molecule studied with encouraging effects in reducing portal hypertension and in improving liver function. However, it has not shown significant changes in the fibrosis stage [242,243].

On the other hand, molecular pathways involved in the innate system’s control have been proposed as effective targets for the treatment of MAFLD and liver fibrosis, since many innate immune cells in the liver offer a first-line defense against organisms and toxins derived from enterohepatic circulation [244]. Nevertheless, liver inflammation and metabolic stress represent a powerful stimulus for the excessive production of inflammation mediators, with consequent hepatocellular damage [158,245]. In order to stop this cascade of events, several clinical trials have been carried out to evaluate the role of ASK1 inhibitors such as Selonsertib (GS-4997) in limiting hepatocellular damage in adult patients with NASH and bridging fibrosis [169,246]. Considering the possibility that additional pathways in the pathogenesis of NASH could bypass the block induced by such inhibitors, other clinical studies have evaluated the potential therapeutical advantages of a combined therapy in adults. A phase 2 clinical trial involving 72 patients with NASH and stage F2–F3 fibrosis has been carried out by treating patients with either 6 or 18 mg Selonsertib orally once daily alone or in combination with a once-weekly injection of 125 mg of Simtuzumab, a humanized monoclonal antibody directed against lysyl oxidase-like molecules. Although all groups showed laboratory, radiological, and histological improvements of the fibrosis stage, the proportion of patients with a decrease of fibrosis of at least one stage at week 24 was greater in patients treated in the 18 mg Selonsertib group than Simtuzumab-alone and the 6 mg Selonsertib group (43%, 20%, and 30%, respectively). These results suggested a possible therapeutic application of a combined strategy [247]. Moreover, since ASK1 plays a fundamental role in responding to external microbial agents, studies on rodents and preclinical models are trying to identify molecules able to modulate its activation via posttranslational modification, such as milk fat globule-epidermal growth factor-8 (MGF-E8), an endogenous inhibitor that halts the progression of hepatic steatosis and inflammation [248,249]. Recently, it has emerged that the loss of intracellular MGF-E8 promotes ASK1 dimerization and phosphorylation in metabolically stressed hepatocytes, thus representing a potentially druggable target [248,249]. Moreover, TNF-alpha-induced protein 3 (TNFAIP3) promotes the deubiquitination of ASK1 in hepatocytes with an anti-inflammatory effect [250]. In this context, TNF receptor-associated factor 6 (TRAF6), which promotes the polyubiquitination of Lys6 connections and the activation of ASK1, could also represent a molecular target to take into account for treating patients with advanced stages of the disease [251].

Similarly to ASK1, TGF-b-activated kinase 1 (TAK1), a member of the MAP3K family with a proinflammatory action, takes part in the pathogenesis of MAFLD and NASH [252,253]. Since TAK1 deletion did not show a histological improvement, the scientific focus has been put on endogenous molecules such as TNFAIP3-interacting protein 3, ubiquitin-specific protease (USP) 4, and USP18, which are described in the literature as negative molecular modulators of liver steatosis, inflammation, and fibrosis able to block enzymatic activity at the posttranslational level [254,255,256,257]. Those first results reported a strong effect of these molecules, but further studies are needed to evaluate their safety and efficacy.

Finally, TLR inhibitors have been the latest pharmacologic strategy studied to treat patient with advanced stages of MAFLD, since TLRs are deeply involved in the pathogenesis of NASH and fibrosis, especially TLR4, which recognizes gut-derived endotoxins [162,258]. Particularly, JKB-121, a TLR4 antagonist, seems to reduce the redox state and HSC activation in the liver [259]. Therefore, a phase 2 study is still in progress to evaluate the applicability of this drug in the clinical setting [260]. In this regard, noting that the gut microbiota is a major source of TLR ligands, it might have a promising future role in treating NAFLD/MAFLD [122,124]. However, the exact mixture of probiotics and/or prebiotics able to limit the damage induced by fat accumulation and oxidative stress is not yet known. Probably, antibiotics, symbiotics (a combination of both a prebiotic and a probiotic), absorbents, anti-inflammatory drugs, and fecal microbiota transplantation can provide support for a lifestyle intervention as a preventive, as well as therapeutic, measure. For instance, rifaximin, a nonabsorbable antibiotic acting on Gram-negative bacteria, reduces proinflammatory cytokine production, showing beneficial effects on patients affected with NAFLD/NASH [261]. In addition, the administration of metformin, beyond its ability to induce weight loss, has led to changes in the gut microbiota composition, favoring the growth of *Bifidobacterium* and *Akkermansia*, mediating a potent anti-inflammatory effect [262]. However, further studies are still needed in order to better define their role in clinical practice.

## 11. Conclusions

Obesity is one of the most severe pathological pictures affecting children of all ages and ethnicity which strongly impacts the risk of MAFLD also in the youngest children. This condition defines a complex and continuous spectrum of histological hepatic damages that strongly affect the development of metabolic alterations. Since a large number of prenatal and postnatal factors are able to influence the occurrence of MAFLD, a deeper understanding of all the molecular pathways behind the insurgence and progression of the disease is needed. The aim is to identify and activate multiple strategies able to change the natural history of the disease and its effects on CVD and metabolic alterations. Although there is not yet a treatment with a proven efficacy for this condition, different medications seem to act as modifiers of liver steatosis, inflammation, and fibrosis. However, further studies, especially in the pediatric population, are urgently needed. Finally, as long as the MAFLD definition, together with its subtypes, is accepted worldwide, it is appropriate to consider the entire spectrum of fatty liver disease as a common outcome pathology with multiple etiological triggers.

## Figures and Tables

**Table 1:** Diagnostic criteria of NAFLD and MAFLD.

NAFLD (Nonalcoholic Fatty Liver Disease)	MAFLD (Metabolic Fatty Liver Disease)
Histological, imaging (ultrasound), or blood biomarker (e.g., ALT) evidence of steatosis	Histological, imaging (ultrasound), or blood biomarker (e.g., ALT) evidence of steatosis
Exclusion of other causes of hepatic steatosis besides NAFLD (e.g., HBV, HCV, drugs, hemochromatosis, autoimmunity, Wilson’s disease, alpha 1 anti-trypsin deficiency, rapid weight loss)	Excess adiposity Presence of prediabetes or Type 2 Diabetes
	Metabolic dysregulation defined by 2 or more altered results on standardized biometric parameters, with a different cut off for each ethnic group *, including: waist circumference, blood pressure triglycerides HDL cholesterol levels fasting glucose triglyceride-to-HDL cholesterol ratio

* values above the 90th, 95th, or 97th percentile for gender and age are used.
